# Robust extraction of biological information from diffusion-weighted magnetic resonance imaging during radiotherapy using semi-automatic delineation

**DOI:** 10.1016/j.phro.2022.02.014

**Published:** 2022-03-07

**Authors:** Anne Louise Højmark Bisgaard, Carsten Brink, Maja Lynge Fransen, Tine Schytte, Claus P. Behrens, Ivan Vogelius, Henrik Dahl Nissen, Faisal Mahmood

**Affiliations:** a Laboratory of Radiation Physics, Department of Oncology, Odense University Hospital, Kløvervænget 19, Indgang 85, Pavillon, stuen, 5000 Odense C, Denmark; b Department of Clinical Research, University of Southern Denmark, Winsløwparken 19, 3. Sal, 5000 Odense C, Denmark; c Department of Radiology, Odense University Hospital, Kløvervænget 47, Indgang 27, 5000 Odense C, Denmark; d Department of Oncology, Odense University Hospital, Kløvervænget 19, 5000 Odense C, Denmark; e Department of Oncology, Herlev and Gentofte Hospital, Borgmester IB Juuls Vej 7, 2730 Herlev, Denmark; f Department of Clinical Oncology, Rigshospitalet, Blegdamsvej 9, 2100 Copenhagen, Denmark; g Department of Clinical Medicine, University of Copenhagen, Blegdamsvej 3B, 2200 Copenhagen N, Denmark; h Department of Oncology, Vejle Hospital, Beriderbakken 4, 7100 Vejle, Denmark

**Keywords:** Diffusion-weighted MRI, Apparent diffusion coefficient, Automatic delineation, MRI guided radiotherapy, Imaging biomarker

## Abstract

**Background and purpose:**

Diffusion-Weighted Magnetic Resonance imaging (DWI) quantifies water mobility through the Apparent Diffusion Coefficient (ADC), a promising radiotherapy response biomarker. ADC measurements depend on manual delineation of a region of interest, a time-consuming and observer-dependent process. Here, the aim was to introduce and test the performance of a new, semi-automatic delineation tool (SADT) for ADC calculation within the viable region of the tumour.

**Materials and methods:**

Thirty patients with rectal cancer were scanned with DWI before radiotherapy (RT) (baseline) and two weeks into RT (week 2). The SADT was based on intensities in b=1100 s mm^−2^ DWI and derived ADC maps. ADC values measured using the SADT and manual delineations were compared using Bland-Altman- and correlation analyses. Delineations were repeated to assess intra-observer variation, and repeatability was estimated using repeated DWI scans.

**Results:**

ADC measured using the SADT and manual delineation showed strong and moderate correlation at baseline and week 2, respectively, with the SADT measuring systematically smaller values. Intra-observer ADC variation was slightly smaller for the SADT compared to manual delineation both at baseline, [−0.00; 0.03] vs. [−0.02; 0.04] 10^−3^ mm^2^ s^−1^, and week 2, [−0.01; 0.00] vs. [−0.04; 0.07] 10^−3^ mm^2^ s^−1^ (68.3% limits of agreement). The ADC change between baseline and week 2 was larger than the ADC uncertainty (±0.04 · 10^−3^ mm^2^ s^−1^) in all cases except one.

**Conclusion:**

The presented SADT showed performance comparable to manual expert delineation, and with sufficient consistency to allow extraction of potential biological information from the viable tumour.

## Highlights

• An intensity-based delineation tool for consistent ADC measurements is presented.
• Semi-automatic delineation performs comparable to manual expert delineation.
• Intra-treatment ADC changes may reflect radiotherapy induced biological changes.

## 1 Introduction

Magnetic Resonance imaging (MRI) is used in radiotherapy (RT) planning as a supplement to computed tomography (CT), primarily because it provides better soft-tissue contrast. Furthermore, advanced MRI techniques can provide information on tumour biology. For example, Diffusion-Weighted MRI (DWI) probes the micro-environment of the tissue by measuring local water mobility [1]. Low water mobility correlates with high cell density, which is often indicative of high tumour viability [2].

DWI is acquired as a set of images with different diffusion weighting, defined through so-called b-values. With higher b-values, the acquisition becomes more sensitive to random motion of water molecules, inflicting large signal loss in regions with high diffusivity. Vice versa, in regions with reduced diffusivity, e.g. due to high cell density, signal loss is small. The water mobility can be quantified if at least two b-values are acquired, as the “Apparent Diffusion Coefficient” (ADC) [2].

ADC is a promising imaging biomarker for treatment response both when derived from pre-treatment imaging and during the course of RT [3], [4], [5]. ADC has also shown potential clinical value in treatment planning and adaptation [6]. However, ADC has yet to be translated into widespread clinical use in RT planning and response evaluation. Lacking randomized trials and lack of consistency in ADC measurement are some of the challenges that need to be addressed before such translation can happen [7], [8].

Recent introduction of the hybrid MRI linear accelerator (MR-linac) has allowed MRI at every treatment fraction, making frequent ADC measurements more accessible [9], [10], [11]. This gives a unique opportunity to collect large data sets to investigate ADC for clinical use. However, to perform large multi-center studies, consistency in ADC calculation and workflow feasibility is important [7].

An important aspect of ADC calculation is the delineation of a region of interest (ROI), which, if done manually, is time-consuming, requires a high level of expertise, and suffers from intra- and inter-observer variation [12], [13]. A potential solution is automated delineation, which is already widely employed in medical images, including standard MRI [14], [15], but not adequately developed for advanced imaging such as DWI. Simple, threshold-based delineation has been tested on DWI, exploiting the large tumour-to-background ratio on raw, high b-value DWI images. Despite promising results, manual inspection was required to avoid non-tumour regions [16]. Fully automated delineation may be achieved using more advanced methods based on artificial intelligence (AI), e.g. convolutional neural networks [17], however, this approach requires training data and suffers from a lack of transparency.

In this study, we present a simple, semi-automatic tool for delineation of the viable tumour volume (VTV), a recommended ROI for ADC measurement, which is not directly identifiable from raw, high b-value DWI images [18], [19], [20]. Instead, the VTV was in this study defined using combined information from both raw, high b-value DWI images and derived ADC maps [20]. The study aim was to test the performance of the tool in terms of robustness of ADC measurements, compared to manual delineation. Also, the capacity of the tool to measure temporal changes in ADC was tested using longitudinal DWI data.

## 2 Materials and methods

### 2.1 Patients

This prospective study included thirty patients with biopsy-proven locally advanced adenocarcinoma of the rectum. All patients received long-course chemoradiotherapy and were treated with a daily fraction five times a week. Tumour was prescribed a dose of 60 Gy in 30 fractions, and the elective lymph node volumes were prescribed 50 Gy in 30 fractions using a concomitant boost intensity-modulated radiation therapy (IMRT) technique. All patients received a 5 Gy brachytherapy boost. The Regional Committee on Health Research Ethics for Southern Denmark has approved the study (study ID S-20110021 and S-20130030), and informed consent was obtained from all patients.

### 2.2 MRI protocol

Patients were MRI scanned before RT (baseline) and two weeks into RT (week 2) with a 1.5 T clinical MRI scanner (Philips Ingenia, Philips Healthcare, Best, The Netherlands). The imaging protocol consisted of T2-weighted imaging (T2W) and DWI. T2W was acquired using a turbo spin-echo sequence (repetition time (TR)/echo time (TE): 7161/100 ms) with an in-plane resolution of (0.8 × 0.8) mm^2^, a slice thickness of 2.4 mm, and a slice gap of 1.0 mm. Scan duration was 5 min and 43 s. DWI was implemented as a single-shot spin-echo echo-planar imaging sequence (TR/TE: 2860/82 ms) with fat suppression (spectral presaturation with inversion recovery) and with b-values ranging from 0 to 1100 s mm^−2^ (0 (2), 7 (2), 20 (2), 40 (2), 90 (2), 170 (2), 300 (2), 500 (4), 700 (4), 900 (4) and 1100 (6) s mm^−2^); with the number of image averages for each b-value given in parenthesis. In this study, only b=0, b=170 and b=1100 s mm^−2^ were used. In-plane resolution was (1.82 × 1.82) mm^2^, slice thickness 4.6 mm and slice gap 0.4 mm. Sequence duration was 4 min and 23 s. In each imaging session, DWI was performed twice in succession (test–retest), while the patient remained in the same position to assess repeatability.

### 2.3 Included data

Thirty patients were MRI scanned at baseline, and out of these patients, twenty-nine were MRI scanned at week 2, resulting in a total of 59 scan sets of images (each set including T2W and DWI for both test and retest). Of these 59 sets, five sets of DWI images were excluded either due to artefacts in DWI (2), severe bulk motion (1), or the tumour being partly outside the field of view (2). In total, full scan data from 27 image sessions at baseline and 27 image sessions at week 2 were used to evaluate repeatability and intra-observer variation at baseline and at week 2. Of these, 25 patients had all scans available at both time-points and were used to assess ADC change between baseline and week 2.

### 2.4 ADC calculation

A set of b-values (170 and 1100 s mm^−2^) were selected for ADC calculation; the high b-value was selected to obtain high diffusion sensitivity and the non-zero, low b-value was selected to avoid perfusion effects [21], [22]. ADC-maps were calculated voxel-wise by applying linear regression to the logarithm of the signal intensity S to get ln(S_high_) = ln(S_low_) - [b_high_-b_low_] · ADC.

### 2.5 Semi-automatic delineation tool

A semi-automatic delineation tool (henceforward named ‘SADT’) was developed with the purpose of delineating VTVs for ADC calculation. VTV was defined as viable tumour, excluding necrotic regions [18], [20]. The SADT was implemented using in-house developed software (Matlab R2019a, Mathworks ab, Sweden), following a 3-step process (Fig. S1 in supplementary matrials).^2^

In step 1, a rough delineation of the relevant 3D region was given as manual input (manual mask). The manual mask indicates the ‘relevant’ region, and the algorithm described below is, except for the very last step, only performed on voxels within this region.

In step 2, 3D thresholding in b=1100 s mm^−2^ DWI and ADC-maps was used to define two masks that fulfilled criteria of high DWI intensity and low ADC values, respectively. In the b=1100 s mm^−2^ DWI image, a binary DWI mask for the bright voxels was created using a threshold value obtained from the Otsu algorithm [23]. Furthermore, an ADC-mask indicating the low ADC values was created from the ADC map using a threshold value equal to the median ADC value plus 0.5 times the standard deviation of the measured ADC values within the manual mask. Half the standard deviation was used to obtain a stable delineation.

In step 3, the overlap between the DWI mask and the ADC mask was created and used as the raw VTV, fulfilling both criteria (high DWI intensity and low ADC values).

The final VTV was obtained by a 2-step post-processing of the raw VTV: First, only the largest volume was retained if non-connected sub-regions were included in the raw VTV. Second, to account for the possibility that the manual mask (Step 1) might have excluded target voxels, the VTV was allowed to expand beyond the boundaries of the manual mask iteratively while respecting threshold criteria (Step 2) and a criterion of connectivity.

### 2.6 Delineation of viable tumour volumes

A radiologist (author MLF) performed manual VTV delineation on b=1100 s mm^−2^ DWI images guided by T2W and ADC-maps on test–retest data for all included patients. The same radiologist re-contoured the images after two months for intra-observer variation assessment. T2W and DWI were rigidly registered prior to delineation using clinical software (MIM, MIM Software Inc., Cleveland, Ohio).

A non-radiologist (author ALHB, physicist, experienced with DWI) used the SADT to delineate the same cases as the radiologist including test–retest data. The manual mask used for input to the SADT included rectum, mesorectum, and some surrounding areas respecting anatomical boundaries to other anatomical structures, e.g. prostate, and was restricted to the same slices as included by the radiologist. Delineation was performed on b=0 s mm^−2^ DWI images using b=1100 s mm^−2^ DWI images for guidance. The same observer created the manual mask input for the SADT twice with a time interval of at least one day to assess intra-observer variation.

### 2.7 Statistics

Measured ADC values were median ADC within the VTV. Bland-Altman analysis [24] was used both at baseline and week 2 to compare delineation methods, and to evaluate intra-observer ADC variation (repeated delineations on the same scan) and ADC repeatability (test–retest difference). As data were non-normally distributed, non-parametric statistics (median, 15.9% and 84.2% percentiles) were used in the Bland Altman analysis to describe bias and 68.3% limits of agreement (LOA) for the observed ADC differences; mimicking a one standard deviation confidence interval (CI) for normal distributed data. Correlation between ADC values from the two delineation methods was assessed using Pearson’s correlation coefficient, including the 95% CI.

To obtain an uncertainty estimate of the ADC values measured with the SADT, all test–retest values at baseline and week 2 were analysed together, bearing in mind that the test–retest differences represented a combined imaging and delineation uncertainty. The uncertainty was estimated as the range between the 15.9% and 84.2% percentiles of the distribution of the differences in median ADC between test and retest. For each individual test–retest scan, ADC differences were calculated as both ‘test minus retest’ and ‘retest minus test’ to get a symmetric distribution with zero mean. The obtained uncertainty estimate was used to evaluate whether a significant change in ADC values between baseline and week 2 could be observed.

## 3 Results

### 3.1 Comparison of semi-automatic and manual delineation

**Fig. 1:**
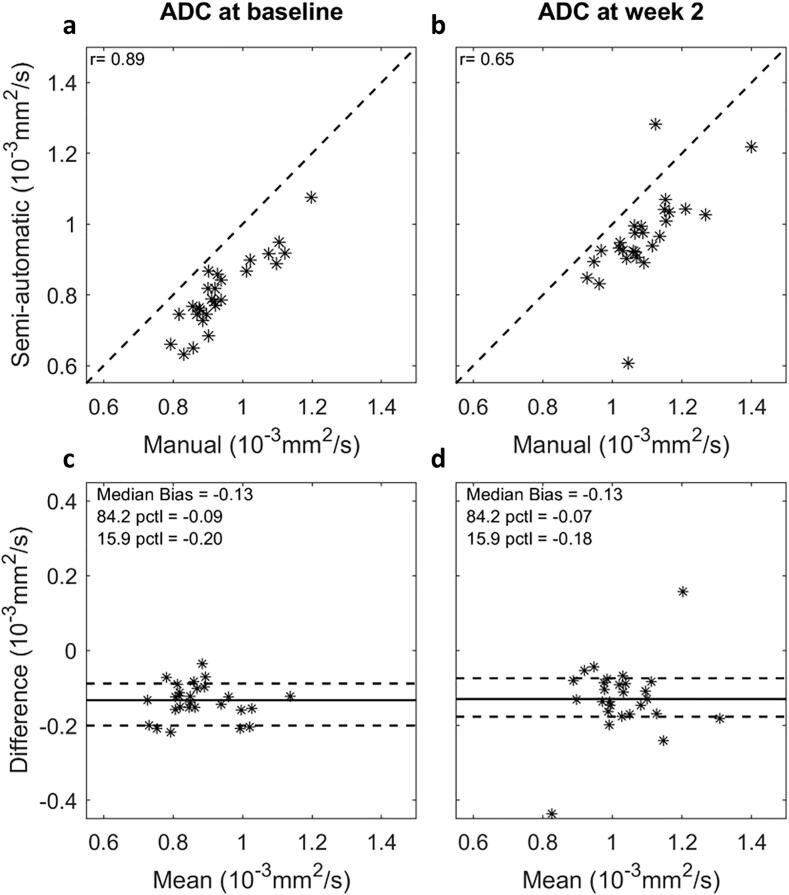
Comparison of delineation methods: Correlation plots (a-b) and Bland-Altman plots (c-d) comparing ADC values measured using semi-automatic delineation by a non-radiologist and manual delineation by a radiologist, at baseline and week 2 in RT. Pearson's correlation coefficient (r) is shown on correlation plots. The Bland-Altman plots show the ADC difference (semi-automatic minus manual) against the mean ADC; the solid and dashed lines represent median ADC difference and 68.3% limits of agreement, respectively. Two extreme measurements were observed at week 2 (−0.437 · 10^−3^ and 0.158 · 10^−3^ mm^2^ s^−1^); the first may be explained by the fact that tumour volume was very small, making ADC calculation sensitive to delineation, and the second by a sub-optimal SADT delineation.

Median ADC values were systematically smaller when derived using the SADT compared to manual delineation by a radiologist. This difference was observed both at baseline and week 2 (Fig. 1). The observed median differences and LOA as given in the method section were −0.13 [−0.20; −0.09] 10^−3^ mm^2^ s^−1^ and −0.13 [−0.18; −0.07] 10^−3^ mm^2^ s^−1^ for baseline and week 2, respectively. The Bland Altman plots in Fig. 1 did not indicate any association between ADC differences and ADC values. The correlation [95% CI] of ADC between the two delineation methods was 0.89 [0.78, 0.95] at baseline and 0.65 [0.36, 0.83] at week 2.

**Fig. 2:**
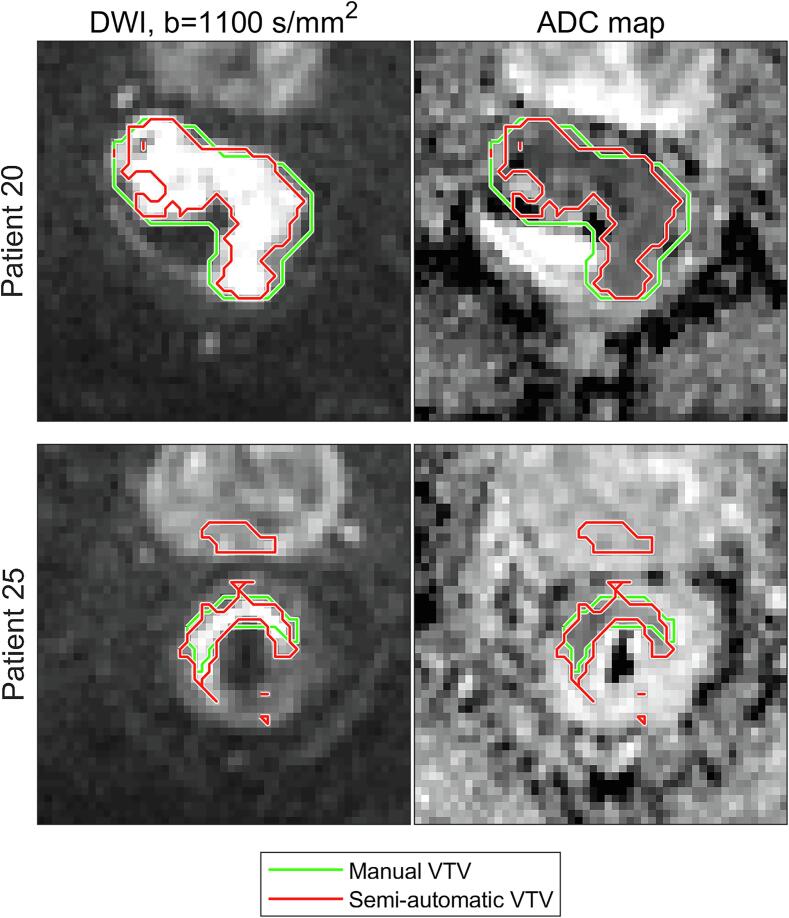
Delineation agreement: Example of a good (Patient 20) and a bad (Patient 25) agreement between manual (green) and semi-automatic (red) delineations for two patients. The images are transaxial and have been cropped, such that an area of (92.8 × 92.8) mm^2^ is shown. Rectum and part of the prostate are visible. Although the VTV defined by the semi-automatic delineation tool (SADT) appears as several separated regions when presented in 2D, it is in fact one connected 3D region. (For interpretation of the references to colour in this figure legend, the reader is referred to the web version of this article.)

By visual inspection, representative examples of good (patient 20) and poor (patient 25) delineation agreements between the SADT and manual delineation were identified (Fig. 2). VTVs delineated with the SADT were typically smaller than manually delineated VTVs (mean volume was 42% smaller at baseline and 39% smaller at week 2).

**Fig. 3:**
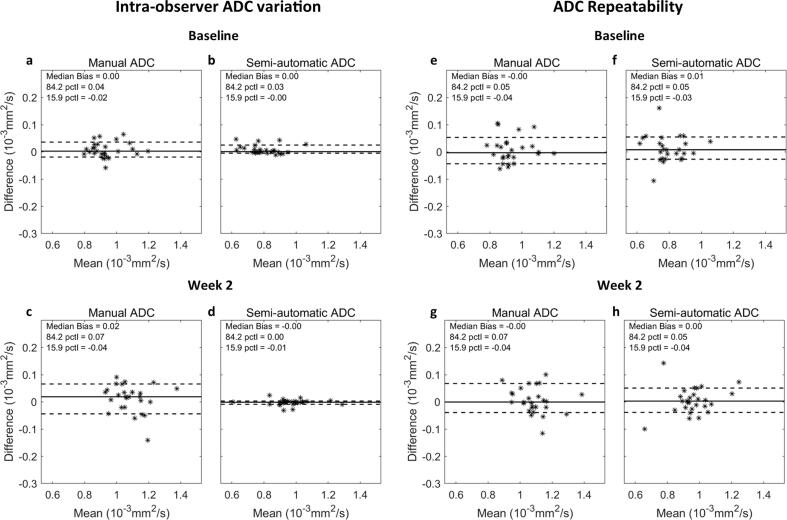
ADC variation: Bland-Altman plots showing intra-observer ADC variation (a-d) and ADC repeatability (e-h) at baseline and week 2 for delineation with the semi-automatic delineation tool (SADT) by a non-radiologist and manual delineation by a radiologist. The solid and dashed lines represent median ADC difference and 68.3% limits of agreement, respectively. The limits of agreement is defined as the 15.9% and 84.2% percentiles (pctl) of the ADC differences.

The intra-observer ADC variation (repeated delineations on the same scan) was compared between the manual delineation and the SADT at baseline (Fig. 3a-b) and week 2 (Fig. 3c-d). For manual delineation, the median difference and LOA were 0.00 [−0.02; 0.04] 10^−3^ mm^2^ s^−1^ at baseline and 0.02 [−0.04; 0.07] 10^−3^ mm^2^ s^−1^ at week 2. For the SADT, the median difference and LOA were 0.00 [−0.00; 0.03] 10^−3^ mm^2^ s^−1^ at baseline and −0.00 [−0.01; 0.00] 10^−3^ mm^2^ s^−1^ at week 2; hence, the tool demonstrated a smaller intra-observer ADC variation compared to manual delineation.

The ADC repeatability (test–retest difference) was compared between manual delineation and the SADT at baseline (Fig. 3e-f) and week 2 (Fig. 3g-h). For manual delineation, the median difference and LOA were −0.00 [−0.04; 0.05] 10^−3^ mm^2^ s^−1^ at baseline and −0.00 [−0.04; 0.07] 10^−3^ mm^2^ s^−1^ at week 2, and for the SADT 0.01 [−0.03; 0.05] 10^−3^ mm^2^ s^−1^ at baseline and 0.00 [−0.04; 0.05] 10^−3^ mm^2^ s^−1^ at week 2. Thus, the ADC repeatability was comparable between the two delineation methods.

### 3.2 ADC changes and related uncertainty

**Fig. 4:**
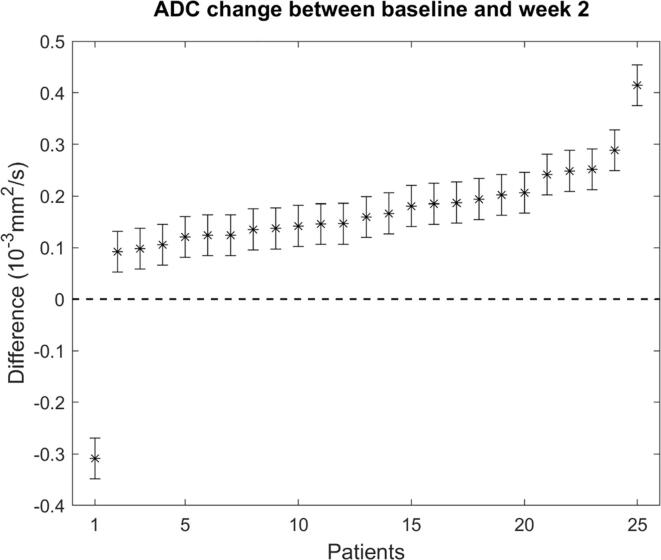
Temporal ADC changes: ADC change between baseline and week 2 measured using the semi-automatic delineation tool (SADT). The error bars represent the estimated ADC uncertainty described in Section 2.7 (±0.04  mm^2^ s^−1^). The ordering of patients on the x-axis is arranged to show increasing ADC change from left to right.

For each patient, the ADC change between baseline and week 2 was evaluated when using the SADT. The obtained differences showed an increase between baseline and week 2 for all patients except one, with a mean ADC increase of 0.159 · 10^−3^ mm^2^ s^−1^ (Fig. 4). The error bars in Fig. 4 represent the ADC uncertainty estimate derived in section 2.7. The ADC increase was larger than the uncertainty (±0.04 · 10^−3^ mm^2^ s^−1^) in all cases.

## 4 Discussion

The SADT for ADC calculation of tumours was implemented to improve consistency of ADC measurements and increase feasibility in the clinical workflow of MR guided RT. The observed correlation between the SADT and manual delineation was strong at baseline and moderate at week 2, with the SADT measuring systematically smaller ADC values. A clearly smaller intra-observer ADC variance was seen for the SADT compared to manual delineation, and ADC repeatability was comparable between the delineation methods. In all patients but one, an ADC change larger than the uncertainty was observed between baseline and week 2 using the SADT. This supported the assumption that ADC might carry potential biological information which can be detected using the presented SADT. The SADT was simple, transparent, easy to implement, and may be used in other types of cancer.

There has been debate as to which ROI strategy to use for ADC calculation [19], [20], [25]. Using the VTV is a recommended strategy, which has been explored in recent studies [18], [19], [20]. One study found that ADC within the VTV was a potential response biomarker and that the VTV was more relevant for response prediction than the GTV [18]. It would be highly relevant to validate the VTV approach against histology in future studies. The presented SADT delineated the VTV based on the underlying assumption that hyperintense areas on high b-value DWI correspond to high cellularity. This assumption has been widely supported [26], although a lower degree of correlation has been observed in patients [27]. A higher correlation might have been found if delineations were better confined to viable tumour sub-regions, such as the VTV. To prevent the SADT from capturing false high cellularity regions, due to so-called T2-shine-through, ADC maps were included in the delineation process, to impose the low-ADC criterion. The SADT made use of Otsu’s method of thresholding to determine a threshold automatically based on the intensity distribution within the manual mask. This approach allowed the threshold to be tailored to a specific ROI in a particular image volume; hence, the threshold differed between delineations. This method was preferred over a constant threshold level due to the arbitrary intensities in MRI and the possibility of intensity variation across images and MRI scanners. A drawback of this method, however, was a slight sensitivity to the manual input.

Some differences between the manually and semi-automatically segmented VTVs were observed (Fig. 2). The manual delineation by the radiologist included more of the periphery of the tumour region, leading to a systematically higher ADC (Fig. 1) since the diffusion usually is less restricted in the surrounding normal tissue. Furthermore, the manual delineations did not have as concave shapes as those created from the SADT, as seen in the upper part of Fig. 2. In general, the SADT seemed to perform as intended, and the observed differences in size and shape between delineation methods were not considered a failure of the SADT. However, in a few cases, VTVs delineated with the SADT erroneously included healthy tissue, e.g. prostate tissue, as illustrated in Fig. 2, patient 25. This error arose due to the expansion of the VTV in the post-processing step of the SADT’s algorithm. However, rejecting the expansion-step might lead to exclusion of relevant regions. This implied that the resulting contours should be reviewed for obvious errors. Overall, the two delineation methods correlated well, indicating that the same tendency could be captured. Since the purpose of the SADT was not to mimic manual delineation but rather to deliver a reproducible measure of a representative tumour ADC, the observed offset between semi-automatic and manual delineation was considered acceptable.

Manual delineation showed larger intra-observer ADC variation at week 2 compared to baseline (Fig. 3). This may be explained by the fact that the tumour volume decreased, and the tumour outline became less clear, as it is often seen during the course of RT. No information of tumour size and position was available to the radiologist during delineation, which might have caused the manual intra-observer variation to be larger than in normal clinical situations. In comparison, the intra-observer variation was smaller for the SADT, showing its potential to improve the consistency of ADC measurements. Potentially, the SADT may also reduce the inter-observer ADC variation, which should be investigated in a follow-up study.

ADC repeatability was affected by imaging-related uncertainty and intra-observer variation. The test–retest scans were acquired in quick succession while the patient remained positioned on the treatment table. Re-positioning the patient between the scans might give a better estimate of the actual clinical ADC repeatability. Though, even without re-positioning, tumour motion (due to bulk motion, peristalsis, gas etc.) between test and retest was evident, and may therefore be a good estimate of the true repeatability. Due to observed differences in tumour position and shape between test and retest, it was found more appropriate to re-contour on test and retest, than to apply structure propagation between scans. This was also representative of a clinical setup where delineations are made on the data by hand.

In all patients except one, ADC increased between baseline and week 2 (Fig. 4). An increase in ADC during RT is in correspondence with earlier ADC studies and might be explained by radiation-induced cellular changes [3], [28]. The observed ADC change was larger than the ADC uncertainty in all cases, indicating a potential biological change. In this study, ADC changes were not compared to treatment response, as the aim was to evaluate the capacity of the SADT to extract potential biological changes and not response prediction in this particular patient cohort.

The manual mask defined by the non-radiologist was restricted to the same slices as included in the radiologist́s delineation. Hence, only the in-plane delineations were compared between the SADT and the expert manual delineation. This was done to allow a more fair comparison of the delineation methods, since the non-radiologist was inexperienced in recognizing rectal tumours, although it limits the use of the SADT as a standalone tool for ADC calculation. Nevertheless, in a clinical workflow, it may be preferable to use the GTV as manual input to the SADT since the VTV is by definition a sub-region of the GTV. The GTV could be obtained by AI based delineation if this became robust and commonly available. In all cases, the GTV as manual input is appealing since GTVs are already available from the normal workflow, and it may facilitate treatment adaption based on ADC in an MRI guided RT workflow on the MR-linac.

In conclusion, the presented SADT showed performance comparable to manual expert delineation, and demonstrated potential to improve consistency of ADC measurements. The SADT was able to detect temporal ADC changes larger than the uncertainty associated with ADC measurements, which implies capability of measuring ADC changes attributed to change in tumour biology during the course of RT. The SADT may therefore prove useful for validation of ADC as a treatment response biomarker.

## Funding

This work was supported by Danish Cancer Society (Grant no. R231-A13852), Danish Comprehensive Cancer Center RT (Danish Cancer Society grant) (Grant no. R191-A11526), and by MANTRA (New MAgNetic resonance Technology for Response Adapted radiotherapy), a Frontline research center based at Odense University Hospital, Denmark. The funding sources had no role in the study design, collection, analysis and interpretation of data, writing of the report or in the decision to submit the article for publication.

## Declaration of Competing Interest

The authors declare that they have no known competing financial interests or personal relationships that could have appeared to influence the work reported in this paper.

## Footnotes

2 The code is available upon request to the corresponding author.
