# Supplementary material for: Robust extraction of biological information from diffusion-weighted magnetic resonance imaging during radiotherapy using semi-automatic delineation
Source: Phys Imaging Radiat Oncol. 2022 Mar 7;21:146–52. doi: 10.1016/j.phro.2022.02.014 (PMC8908275; doi:10.1016/j.phro.2022.02.014)
Supplement: Supplementary data 1 [file mmc1.pdf]

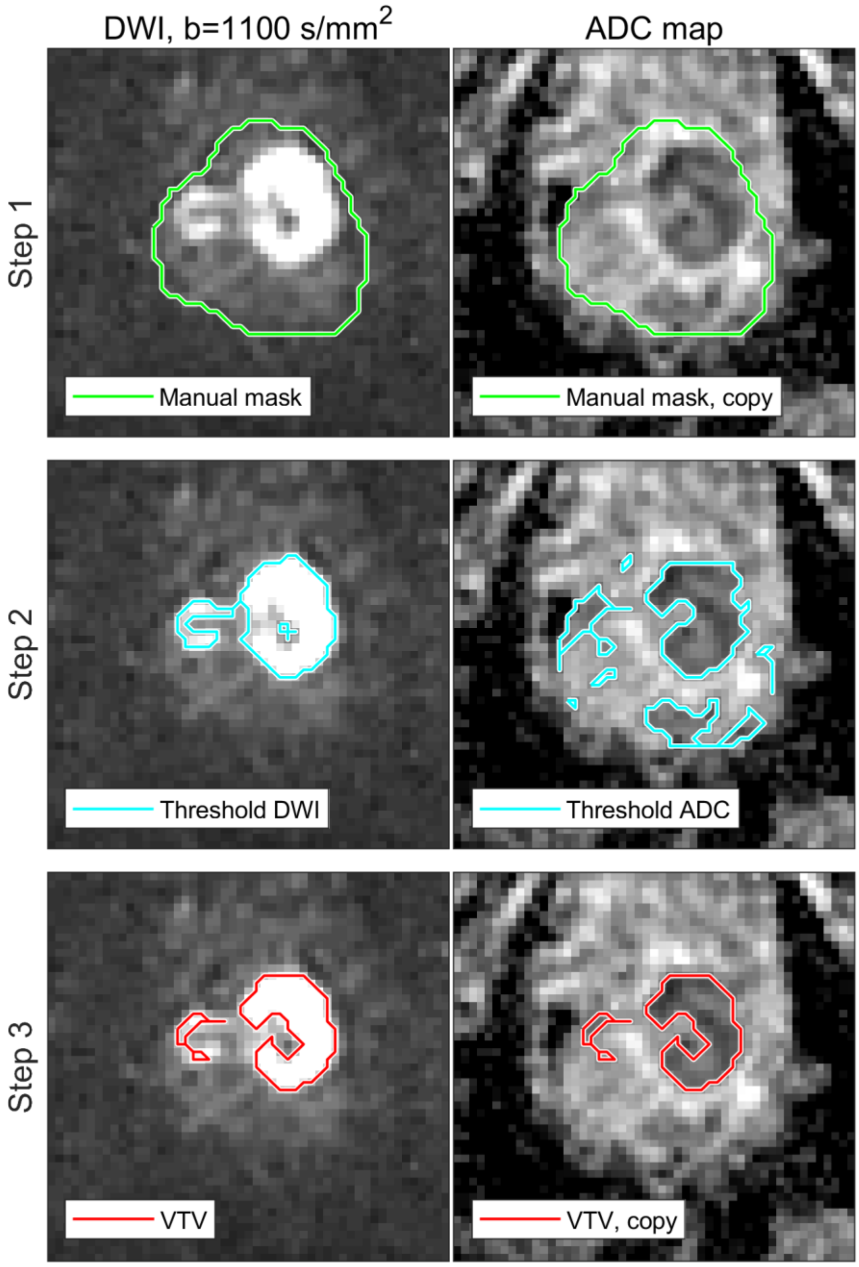

Figure S1. 3-step process of the semi-automatic delineation tool (SADT): Step 1: manual input delineates roughly the region of interest, Step 2: two masks are created based on criteria of high DWI intensity and low ADC, Step 3: overlap between the masks from step 2 defines the resulting VTV. In this example, the manual mask was defined by the non-radiologist. The image is transaxial and has been cropped to a size of (92.8x92.8) mm^2. Note that semi-automatic delineation is performed on 3D images.
